# The effect of tauroursodeoxycholic Acid (TUDCA) treatment on placental endoplasmic reticulum (ER) stress in a rat model of advanced maternal age

**DOI:** 10.1371/journal.pone.0282442

**Published:** 2023-04-06

**Authors:** Mazhar Pasha, Raven Kirschenman, Amy Wooldridge, Floor Spaans, Christy-Lynn M. Cooke, Sandra T. Davidge

**Affiliations:** 1 Department of Physiology, University of Alberta, Edmonton, Alberta, Canada; 2 Department of Obstetrics and Gynecology, University of Alberta, Edmonton, Alberta, Canada; 3 Women and Children’s Health Research Institute, University of Alberta, Edmonton, Alberta, Canada; University of Abuja Teaching Hospital, NIGERIA

## Abstract

Advanced maternal age (≥35 years) is associated with an increased risk of pregnancy complications such as fetal growth restriction and preeclampsia. We previously demonstrated poor pregnancy outcomes (reduced fetal body weight), altered vascular function, and increased expression of endoplasmic reticulum (ER) stress markers (phospho-eIF2α and CHOP) in mesenteric arteries from a rat model of advanced maternal age. Further, treatment of aged dams during pregnancy with an ER stress inhibitor, tauroursodeoxycholic acid (TUDCA) increased fetal body weight (both male and female), tended to improve uterine artery function, and reduced expression of phospho-eIF2α and CHOP in systemic arteries. Placental ER stress has been linked to poor pregnancy outcomes in complicated pregnancies but whether placental ER stress is evident in advanced maternal age is not known. In addition, sex-specific changes in the placental labyrinth and junctional zones from male and female offspring in advanced maternal age have not been investigated. Therefore, the current study aimed to investigate the effect of TUDCA intervention on placental ER stress. We hypothesize that placental ER stress is increased in a rat model of advanced maternal age that is alleviated by TUDCA intervention for both sexes. Placental ER stress markers (GRP78, phospho-eIF2α, ATF-4, CHOP, ATF-6α, and sXBP-1) were quantified by Western blot in placentas from male and female offspring; the labyrinth and junction zones were analyzed separately. In the placental labyrinth zone from male offspring, only GRP78 (p = 0.007) was increased in aged dams compared to young dams; TUDCA treatment reduced the placental expression of GRP78 in aged dams (p = 0.003). In addition, TUDCA reduced the levels of phospho-eIF2α (p = 0.021), ATF-4 (p = 0.016), and CHOP (p = 0.012) in aged dams but no effect was observed in young TUDCA-treated dams. In the placental labyrinth zone from female offspring, an increased level of phospho-eIF2α (p = 0.005) was observed in aged dams compared to young dams, and TUDCA treatment had no effect in both young and aged groups. In the placental junctional zone from male and female offspring, no changes in the expression of GRP78, phospho-eIF2α, ATF-4, CHOP, and ATF-6α was observed with or without TUDCA treatment in both young and aged groups, however, a reduced expression of sXBP-1 protein was observed in from both male (p = 0.001) and female (p = 0.031) placentas from aged-TUDCA treated dams compared to aged control. In conclusion, our data highlight the complexity and sex-specificity of ER stress responses in advanced maternal age with TUDCA treatment maintaining ER stress proteins to basal levels and improving fetal growth in both male and female offspring.

## Introduction

Advanced maternal age is generally defined as maternal age ≥35 years during the time of delivery. Studies revealed that with advancing age there is an increased risk of pregnancy-related complications including fetal growth restriction, gestational hypertension, diabetes, preterm birth, preeclampsia, and stillbirth [1–6]. Previously, in a rat model of advanced maternal age, we have observed poor pregnancy outcomes (reduced fetal body weight, increased placental weight, reduced number of pups, and increased resorptions rate) and altered vascular function (enhanced nitric oxide and endothelium-dependent hyperpolarization) in mesenteric arteries in aged dams compared to young dams [7, 8]. However, the molecular mechanisms associated with poor pregnancy outcomes and altered vascular function with advancing age remains an area of active scientific investigation.

At the molecular level, several studies have highlighted the contribution of endoplasmic reticulum (ER) stress leading to vascular dysfunction in aging vessels (non-pregnant state) [9–13] and poor pregnancy outcomes in complicated pregnancies [14, 15]. ER is an important organelle within the cell that is responsible for protein folding and post-translational modification, and any disturbance within the cell or normal ER function leads to the accumulation of misfolded or unfolded proteins resulting in ER stress. Subsequently, activating the highly conserved cellular adaptive response pathway known as the unfolded protein response (UPR). UPR is mediated by three main key sensor proteins, inositol-requiring enzyme-1α (IRE1α), PKR-like ER kinase (PERK), and activating transcription factor 6α (ATF6α). The main purpose of UPR is to restore ER homeostasis by increasing the synthesis of the molecular chaperones that helps in protein folding and/or degradation of misfolded proteins, however, prolonged accumulation of unfolded proteins may activate the apoptotic pathway. Thus, ER stress has the capacity to decide cell fate via inducing the survival pathway or programmed cell death [9, 11, 16–19].

Indeed, in a rat model of advanced maternal age, previously, we have observed increased expression of ER stress markers (phospho-eIF2 alpha and CHOP) in mesenteric arteries from aged dams compared to young dams, and through an intervention study using tauroursodeoxycholic acid (TUDCA; an ER stress inhibitor) we demonstrated reduced vascular expression of ER stress marker (phospho-eIF2alpha and CHOP proteins), improved fetal body weight, and a tendency to improve uterine artery function in aged TUDCA-treated dams compared to control aged dams [20]. TUDCA is a naturally occurring water soluble bile acid that is used to treat cholestasis. Moreover, studies have highlighted that TUDCA prevents ER stress and improves endothelial dysfunction in hypertension and diabetic mouse models [21–23]. However, the potential impact of TUDCA intervention during pregnancy on placental ER stress in advanced maternal age is unknown.

The placenta is a multifunctional organ that plays a pivotal role in fetal growth and development, ensuring a healthy pregnancy outcome. The placenta is arguably the foremost important organ that develops during pregnancy, but paradoxically, understanding the role of the placenta in high-risk pregnancies such as advanced maternal age remains elusive. In the current study, we used a rat model of advanced maternal age as a translational model. Both rat and human placenta have similarities (discoid and hemochorial placentas) with deep trophoblast invasion [24, 25]. The rat placenta is divided into the labyrinth zone and the junctional zone. The labyrinth zone is comprised of both fetal and maternal vasculature and it is the major site having high metabolic activity and is involved in the exchange of nutrients, oxygen, and waste products between the mother and the developing fetus. Whereas, the junctional zone constitutes the main endocrine compartment of the placenta, producing several key hormones, growth factors, and cytokines. Thus, both zones play an important role in regulating maternal and fetal physiology for the maintenance of healthy pregnancy, and normal fetal growth and development [26–29].

Previously, our group has demonstrated increased levels of placental oxidative stress in male and female placentas and elevated levels of apoptosis only in the male placenta of aged dams compared to young dams, indicating that there may be sex differences in the mechanisms behind placental dysfunction in aged dams [30]. However, a role for placental ER stress has not been evaluated; therefore, the current study is a complementary study to evaluate the effect of TUDCA on placental ER stress in the male and female offspring labyrinth and junctional zones. We hypothesize that placental ER stress is increased in a rat model of advanced maternal age that is alleviated by TUDCA intervention for both sexes.

## Materials and methods

### Ethical approval

All experimental methods/procedures were approved by the University of Alberta Health Sciences Animal Policy and Welfare Committee, in accordance with the guidelines of the Canadian Council on Animal Care and the Guide for the Care and Use of Laboratory Animals published by the US National Institutes of Health (AUP #3693).

### Animal model and experimental design

Both male (for breeding) and female Sprague Dawley (SD) rats were purchased from Charles River Canada (St-Constant, QC) at 3 months of age and were housed at an ambient temperature of 22±1°C and a 14:10 h light: dark cycle in the Animal Care Facility at the University of Alberta. An established and characterized rat model of advanced maternal age was used [8, 31, 32], briefly, young pregnant rats were 3–4 months of age (≈18 years of age in humans) [33] and aged pregnant rats were 9–10 months of age corresponding to ≈35 years of age in humans (considering milestones: reproductive senescence weaning, sexual maturity, and skeletal maturity) [31, 34]. Young rats were provided *ad libitum* standard rat chow, whereas aged rats were maintained on a restricted diet of 6 pellets/day based on National Research Council recommendations (to prevent age-related obesity as a confounding factor [35]. Once pregnancy was confirmed by the presence of sperm in a vaginal smear (considered as gestational day GD 0), all rats (control or TUDCA-treated) were fed an *ad libitum* standard chow diet [8, 31]. TUDCA- was provided through the drinking water from GD0-GD20; to a calculated dose of ~150 mg/kg/day [21, 36, 37]. On GD 20 (term = 21–22 days), rats were anesthetized using isoflurane and euthanized by exsanguination via cardiac punction. Pregnancy outcomes such as resorption sites, number of pups, and fetal biometrics including body weight and crown-rump length (CRL), abdominal girth (AG), and placental weight were recorded. Following which, male and female sex in the fetuses were identified by ano-genital distance and corresponding placental labyrinth and junctional zones were separated and snap-frozen for further analysis (Western blotting).

### ER stress markers using Western blot analysis

Frozen placental samples (male and female labyrinth and junctional zones from young/aged control or TUDCA-treated) were homogenized using lysis buffer (concentration in mmol/L: 20 Tris (pH 7.4), 100 sodium, 10 sodium pyrophosphates tetrabasic, 5 EDTA, and 9-fluoride with 1% Nonidet P-40) containing phosphatase inhibitor (2 mmol/L sodium orthovanadate, Sigma), protease inhibitor cocktail (Thermo Scientific), and 1 mmol/L Phenylmethylsulfonyl fluoride (PMSF; Fluka Biochemika). 50μg of protein were loaded and separated on SDS-polyacrylamide gels (10 and 12%) and transferred to a nitrocellulose membrane (100V, 1.5 hours; 0.2μm, Bio-Rad). For normalization, total protein quantification was performed using LI-COR Revert 700 Total Protein Stain and imaged (LI-COR Odyssey system). Following the reversal of the total protein staining, membranes were incubated with Blocking Buffer (Rockland, PA, USA) for 1 hour. Membranes were then incubated overnight at 4°C with primary antibodies for GRP78 (1:1000 rabbit polyclonal, Cell Signaling Technology, Danvers, MA, USA), phospho-eIF2α (1:500 rabbit polyclonal, Cell Signaling Technology), Total-eIF2α (1:500 mouse monoclonal, Santa Cruz Biotechnology), CHOP (1:500 mouse monoclonal, Cell Signaling Technology), sXBP1 (1:500 rabbit polyclonal, Cell Signaling Technology/Proteintech), ATF-4 (1:500 rabbit polyclonal, Proteintech) or ATF-6α (1:1000 mouse monoclonal, Santa Cruz Biotechnology) in phosphate-buffered saline with Tween-20 (PBST in mmol/L; 10 Na_2_HPO_4_, 2.7 KCl, 137 NaCl, 1.8 KH_2_PO_4_, and Tween^®^ 20: 0.1% (w/v); Thermo Scientific). The following day, membranes were incubated with the corresponding secondary antibody: IRDye donkey anti-rabbit IgG (for GRP78, phospho-eIF2α, sXBP-1, and ATF-4) and IRDye donkey anti-mouse IgM (for total-eIF2α, CHOP, and ATF-6α) at 1:10,000 in PBST buffer (LI-COR Biosciences, Lincoln, NE). Finally, blots were visualized with an LI-COR Odyssey Bioimager (LI-COR Biosciences) and quantified using ImageStudioLite software (LI-COR Biosciences). All data were normalized to total protein (except phospho-eIF2α, which was normalized to total-eIF2α) and expressed as % change compared to the respective control group (young pregnant rats).

### Statistical analyses

Data was analyzed by two-way ANOVA with Sidak’s post-test using GraphPad Prism 9 (GraphPad Software, San Diego, CA, USA) and presented as mean ± SEM. p < 0.05 was considered statistically significant. Based on power calculations from previous research/published manuscripts from our lab, using estimates of effect size and standard deviation, with an 80% power and an ⍺-error of 0.05, for the current study we required a sample size of 6 rats/group.

## Results

### TUDCA improved male and female fetal body weight in aged dams

**Fig 1:**
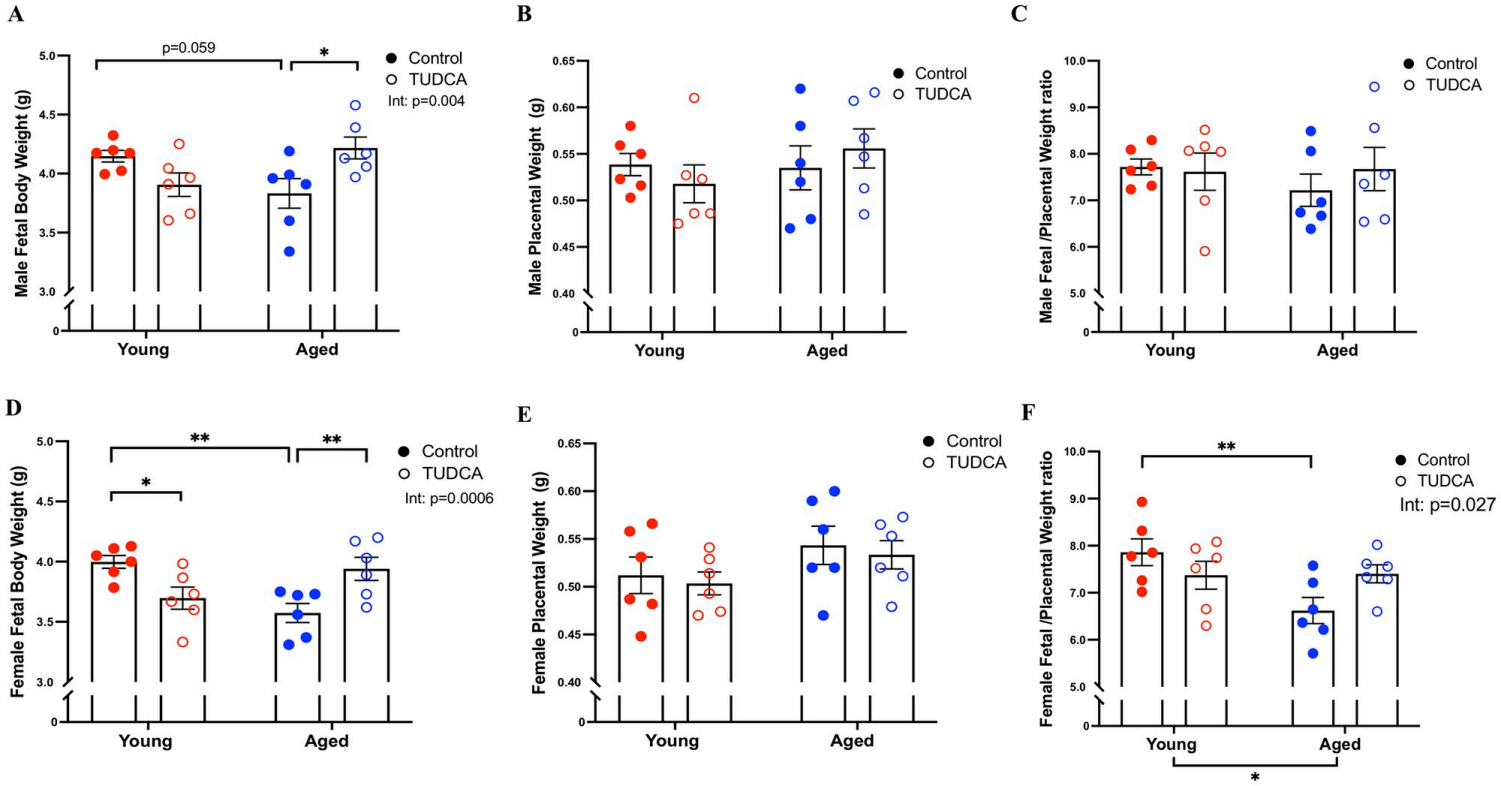
Pregnancy outcomes. Pregnancy outcomes such as **(A, D)** fetal body weight, **(B, E)** placental weight, **(C, F)** fetal/placental weight ratio, in young control (3–4 months; in red) and aged dams (9–10 months; in blue) with or without TUDCA-treatment (open squares) on gestational day 20. Data are presented as mean±SEM; analyzed by two-way ANOVA with planned contrast *post-hoc* test; *p<0.05, **p<0.01; n = 6/group.

Fetal weight was reduced in male (p = 0.059) and female (p<0.01), offspring from aged dams compared to young control dams and while TUDCA treatment improved both male (p<0.05) and female (p<0.01) fetal weight in the aged dams, with no effect in the young dams (Fig 1A and 1D). There was no significant difference in crown-rump length/abdominal girth (CRL/AG) among the groups for either sex (data not shown). Placental weights were not altered by either age or TUDCA-treatment in either sex (Fig 1B and 1E). Fetal/placental weight ratio was similar in all groups in the male offspring (Fig 1C). In females, the fetal/placental weight ratio (p<0.01) was reduced in aged dams compared to young dams, but this was not observed in the TUDCA-treated groups (significant interaction p = 0.027; Fig 1F).

### Male labyrinth zone

**Fig 2:**
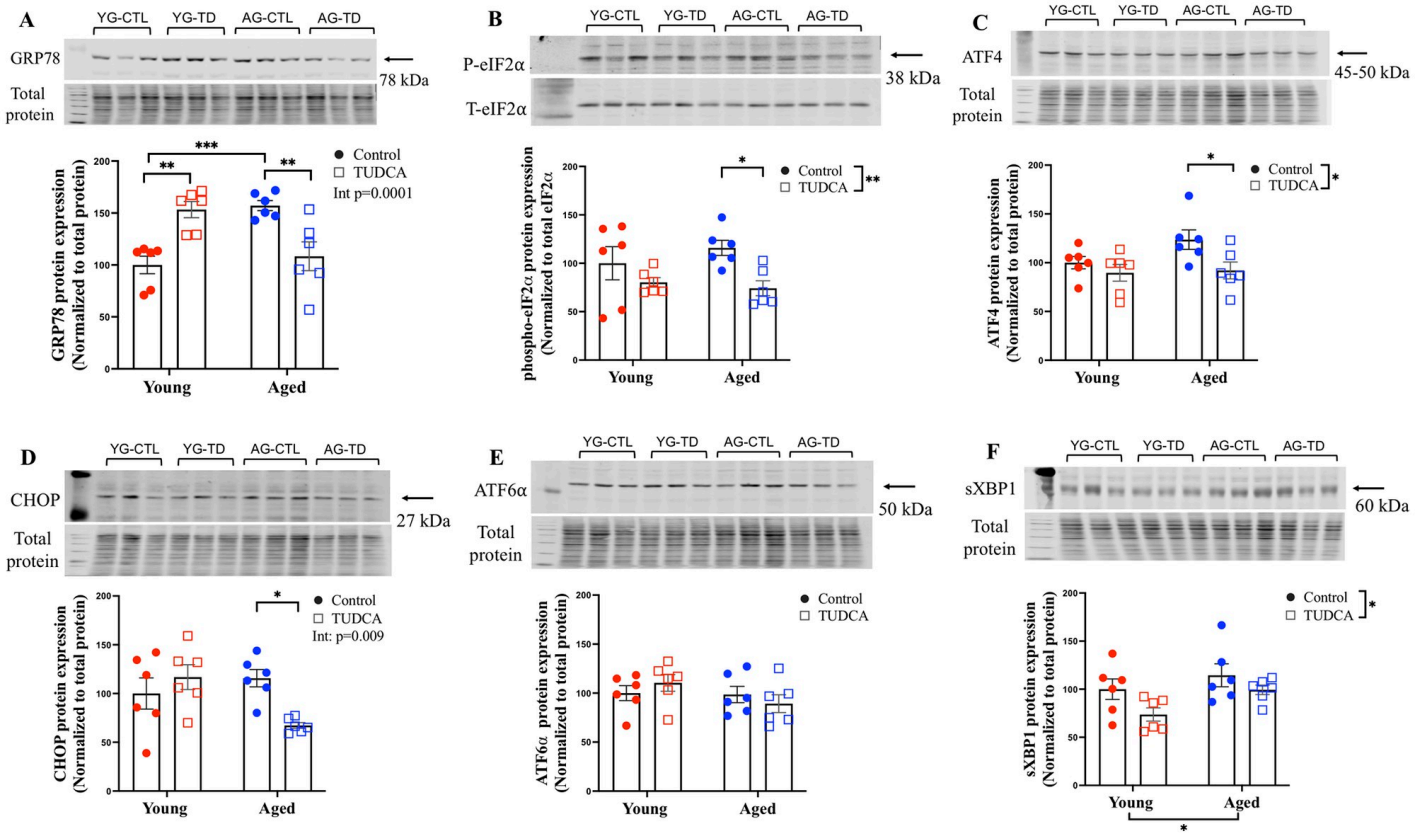
Male labyrinth zone. Western blot analysis of male offspring labyrinth zone (A) GRP78, (B) phospho-eIF2 α, (C) ATF-4, (D) CHOP, (E) ATF-6α, and (F) sXBP-1 normalized to total protein (except phospho-eIF2α normalized to Total eIF2α) in male placenta labyrinth zone of young (3–4 months; in red) and aged control dams (9–9.5 months; in blue) with (open squares) or without (closed circles) TUDCA-treatment on gestational day 20. Data are presented as mean±SEM and expressed as percentage of control (i.e., the mean of the young control dams); analyzed by two-way ANOVA with Sidak’s multiple comparisons post-hoc test; *p<0.05, **p<0.01, ***p<0.001; n = 6/group. YG-CTL = Young control dams; YG-TD = Young TUDCA-treated dams; AG-CTL = Aged control dams; AG-TD = Aged TUDCA-treated dams.

We examined the labyrinth zone of placentas from male offspring and quantified various ER stress markers. Our data demonstrate that there is an increased expression of GRP78 in placentas from aged control dams compared to young control dams (p<0.001). TUDCA treatment significantly reduced the placental expression of GRP78 in aged TUDCA-treated dams compared to aged control (p<0.01) but increased levels of GRP78 in young TUDCA-treated dams vs young control (p<0.0l; Fig 2A). In addition, TUDCA treatment reduced placental expression of phospho-eIF2α, ATF-4, and CHOP in only the aged dams (p<0.05; Fig 2B–2D). No changes in the expression of ATF-6α and sXBP-1 proteins were observed in male placentas of both young and aged control and TUDCA-treated dams (Fig 2E and 2F).

### Female labyrinth zone

**Fig 3:**
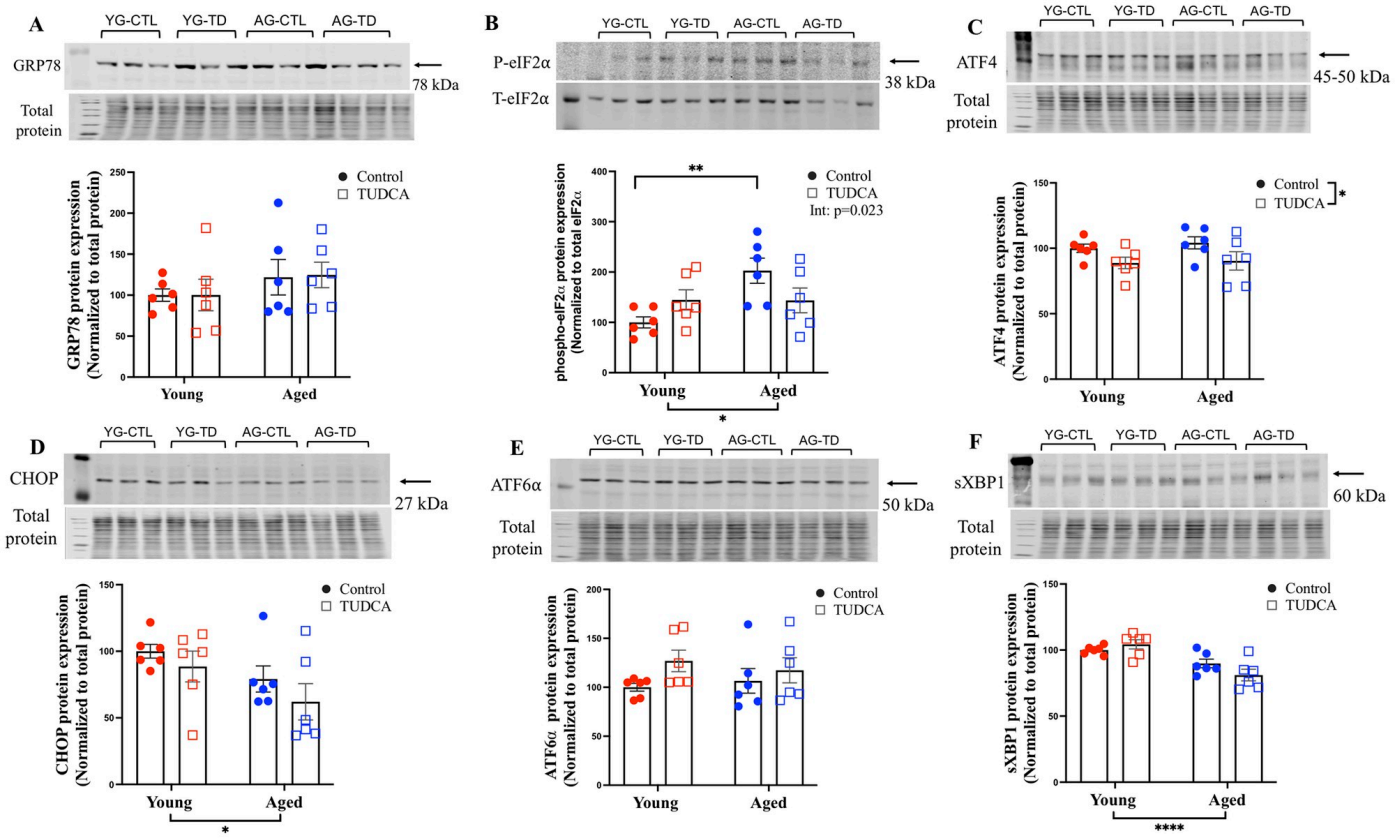
Female labyrinth zone. Western blot analysis of female labyrinth zone (A) GRP78, (B) phospho-eIF2α, (C) ATF-4, (D) CHOP, (E) ATF-6α, and (F) sXBP-1 normalized to total protein (except phospho-eIF2α normalized to Total eIF2α) in female placenta labyrinth zone of young (3–4 months; in red) and aged control dams (9–9.5 months; in blue) with (open squares) or without (closed circles) TUDCA-treatment on gestational day 20. Data are presented as mean±SEM and expressed as percentage of control (i.e., the mean of the young control dams); analyzed by two-way ANOVA with Sidak’s multiple comparisons post-hoc test; *p<0.05, **p<0.01, ****p<0.0001; n = 6/group. YG-CTL = Young control dams; YG-TD = Young TUDCA-treated dams; AG-CTL = Aged control dams; AG-TD = Aged TUDCA-treated dams.

In contrast to placentas from male offspring, in the placental labyrinth zone from female offspring, no changes in the expression of GRP78 were observed in either the young or aged groups (with or without TUDCA treatment) (Fig 3A). Placental expression of phospho-eIF2α was significantly higher in aged control dams vs young control dams (p<0.01) but this was not observed in the TUDCA-treated group (significant interaction p = 0.023; Fig 3B). No changes were seen in the expression of ATF-4, CHOP, ATF-6α, and sXBP-1 in female placentas from both young and aged control and TUDCA-treated dams (Fig 3C–3F).

### Male junctional zone

**Fig 4:**
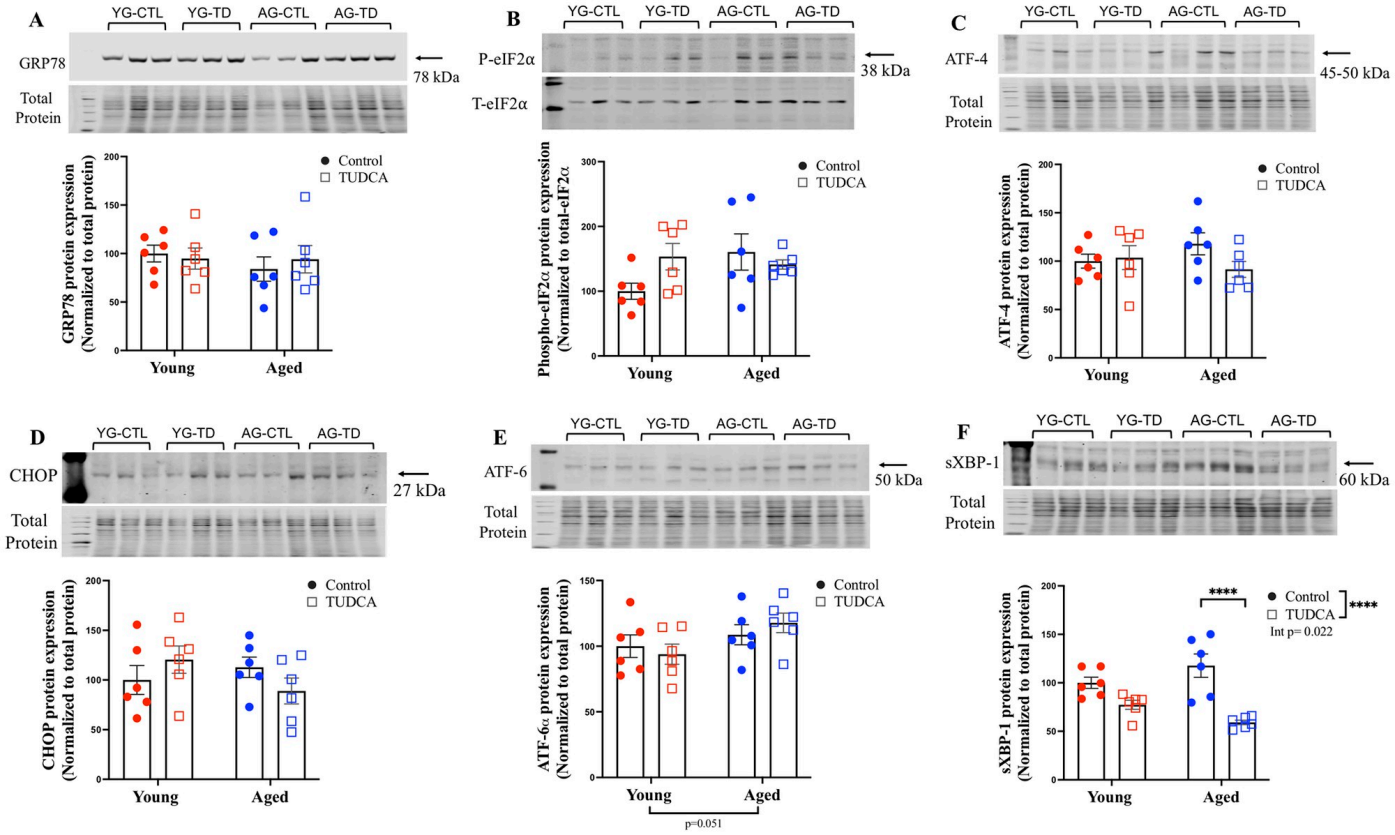
Male junctional zone. Western blot analysis of male junctional zone (A) GRP78, (B) phospho-eIF2α, (C) ATF-4, (D) CHOP, (E) ATF-6α, and (F) sXBP-1 normalized to total protein (except phospho-eIF2α normalized to Total eIF2α) in male placenta junctional zone of young (3–4 months; in red) and aged control dams (9–9.5 months; in blue) with (open squares) or without (closed circles) TUDCA-treatment on gestational day 20. Data are presented as mean±SEM and expressed as percentage of control (i.e., the mean of the young control dams); analyzed by two-way ANOVA with Sidak’s multiple comparisons post-hoc test; ****p<0.0001; n = 6/group. YG-CTL = Young control dams; YG-TD = Young TUDCA-treated dams; AG-CTL = Aged control dams; AG-TD = Aged TUDCA-treated dams.

For the junctional zone of the placenta, no changes in the expression of GRP78, phospho-eIF2α, ATF-4, CHOP, and ATF-6α in male offspring placentas were observed between young and aged groups (with or without TUDCA treatment; Fig 4A and 4C–4E). TUDCA treatment significantly reduced the protein expression of sXBP-1 in male offspring placentas from only the aged dams (p<0.0001; Fig 4F).

### Female junctional zone

**Fig 5:**
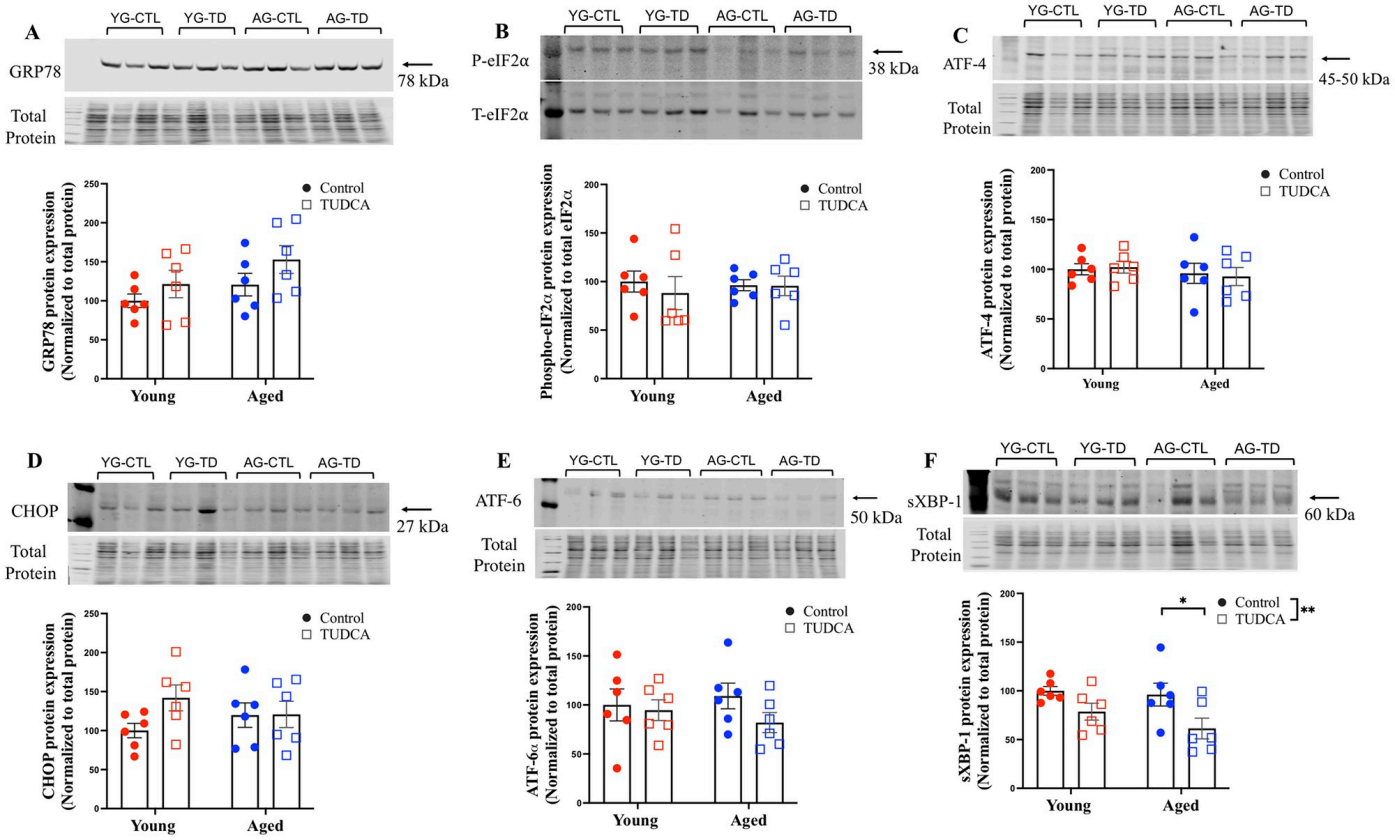
Female junctional zone. Western blot analysis of female junctional zone (A) GRP78, (B) phospho-eIF2α, (C) ATF-4, (D) CHOP, (E) ATF-6α, and (F) sXBP-1 normalized to total protein (except phospho-eIF2α normalized to Total eIF2α) in female placenta junctional zone of young (3–4 months; in red) and aged control dams (9–9.5 months; in blue) with (open squares) or without (closed circles) TUDCA-treatment on gestational day 20. Data are presented as mean±SEM and expressed as percentage of control (i.e., the mean of the young control dams); analyzed by two-way ANOVA with Sidak’s multiple comparisons post-hoc test; *p<0.05; n = 6/group. YG-CTL = Young control dams; YG-TD = Young TUDCA-treated dams; AG-CTL = Aged control dams; AG-TD = Aged TUDCA-treated dams.

For the junctional zone of the placenta, no changes in the expression of GRP78, phospho-eIF2α, ATF-4, CHOP, and ATF-6α in female offspring placentas were seen between young and aged groups (with or without TUDCA treatment; Fig 5A–5E). Similar to the male placentas, TUDCA treatment reduced the expression of sXBP-1 in female offspring placentas from only the aged dams (p<0.05; Fig 5F).

## Discussion

Previously, we observed poor pregnancy outcomes, altered vascular function, and increased ER stress in mesenteric arteries from aged dams, while TUDCA treatment reduced blood pressure, improved fetal body weight (fetal sex was not assessed), tended to improve uterine artery function, and reduced ER markers in mesenteric arteries (phospho-eIF2α and CHOP) in aged dams compared to aged control. These findings suggest a potential beneficial role of TUDCA in advanced maternal age pregnancies [20]. Given the central role of the placenta in regulating fetal growth and overall pregnancy outcomes, we assessed the effect of TUDCA intervention on placental ER stress in both male and female offspring labyrinth and junctional zones in a rat model of advanced maternal age. TUDCA treatment reduced ER stress markers (GRP78, phospho-eIF2α, ATF-4, and CHOP) in the male labyrinth zone and sXBP-1 in the male and female junctional zones without any effect in the female labyrinth zone, suggesting sex differences in placental response with TUDCA intervention. TUDCA also improved fetal body weight in both sexes (that was reduced in the aged dams) without changes in the placental weight in the aged dams. No changes in the fetal weight/placental weight ratio were observed in male fetuses, while reduced fetal weight/placental weight was seen in female fetuses from aged control dams compared to young control dams, suggesting advanced maternal age significantly reduced the placental efficiency in female fetuses, and this aging effect was no longer significant in the TUDCA treated aged dams.

These data suggest a sex-specific reduction in placental efficiency in female offspring from aged dams that was ameliorated by TUDCA treatment.

Studies have highlighted that placental insufficiency (inadequate supply of nutrients and oxygen to the fetus) is the most common cause of fetal growth restriction [38–40]. In general, the supply of nutrients and oxygen occurs through several mechanisms (simple diffusion, pinocytosis, facilitated diffusion, and or via active transporter systems, such as glucose and amino acid transport system [40–43]. Indeed, studies revealed that increased ER stress can impact some of the key nutrient-sensing proteins (mammalian targets of rapamycin and O-GlcNAc transferase pathways) that are involved in amino-acid and glucose transport causing fetal growth restriction [44–46]. TUDCA may indirectly control these nutrient sensors in aged dams to improve fetal weight, however, this remains to be explored in future research.

Placental ER stress has been highlighted in the pathophysiology of fetal growth restriction. For example, Kawakami *et al*. revealed that pregnant mice exposure to prolonged ER stress (tunicamycin) altered the formation of the placental labyrinth zone and induced fetal growth restriction and preterm birth [47]. However, the role of placental ER stress in advanced maternal age is not known. In general, under conditions of ER stress, the master regulator GRP78 is released from the three key UPR sensor proteins (PERK, ATF-6α, and IRE1α) leading to the activation of downstream pathways to restore protein homeostasis. One way of achieving homeostasis is by phosphorylation of eIF2α (phospho-eIF2α via the PERK pathway) which reduces the global protein synthesis of protein, thus, reducing the ER workload. However, if ER fails to regain proteostasis or under the conditions of prolonged ER stress, there is paradoxical activation of ATF-4 and CHOP (a pro-apoptotic protein) eventually leads to apoptosis. In addition, upregulation of sXBP-1 via IRE1α and /or ATF6α promotes cell survival [9, 11, 16, 48].

In the male offspring labyrinth zone, we observed a higher expression of GRP78 protein in aged dams compared to young dams and TUDCA treatment reduced the expression of GRP78 in aged dams vs aged control. The observed up-regulation of GRP78 in aged dams is in line with aging literature [49–51], and in sera (using GRP78 ELISA Kit) and placentae (using immunohistochemistry and Western blotting) of intrauterine growth restriction and early onset preeclampsia [52, 53]. It is known that pregnancy is a stress factor culminating in acute/mild ER stress and one way to respond to this stress is via upregulation of GRP78. In contrast, under excessive or chronic ER stress, initially, upregulation of GRP78 activates the UPR pro-survival pathway, but eventually activates pro-apoptotic protein such as CHOP via the PERK pathway or JNK via IRE1α. This could have been the case with aged dams (increased GRP78), and TUDCA treatment significantly reduced the expression of GRP78. Additionally, TUDCA reduced the expression of phospho-eIF2α, ATF-4 and CHOP only in aged dams compared to aged control, but no changes were seen in young TUDCA-treated dams vs young control. Thus, we may postulate that TUDCA is acting as a protective agent to prevent ER stress by maintaining these proteins (GRP78, phospho-eIF2α, ATF-4, and CHOP) at basal levels in aged dams. Although, not in pregnancy, several studies have demonstrated the cytoprotective effect of TUDCA not only by alleviating ER stress but also by preventing apoptosis [21, 54–58].

In contrast to the aged dams, TUDCA treatment increased the levels of GRP78 in the male (but not female) labyrinth zone in young TUDCA-treated dams compared to young control. As highlighted previously, low-grade ER stress is common in normal pregnancy, and one way to respond to this stress is via upregulation of GRP78, which was evident in young TUDCA-treated dams. Thus, induction of GRP78 could act as a pro-survival arm of the UPR and also as a molecular chaperone regulating protein homeostasis. Supporting this notion, Luo *et al*. demonstrated that mouse embryos with the knockout of GRP78 showed reduced proliferation, apoptosis, and embryonic lethality [59]. In the current study, while the exact mechanism is not clear, we speculate that TUDCA might work differentially under conditions of low-grade vs severe ER stress. Indeed, emerging studies have demonstrated that low-grade ER stress could be beneficial by eliciting an adaptive UPR response that preconditions to a subsequent harmful insult (a condition known as ER hormesis) [60–64]. Thus, TUDCA likely increases the levels of molecular chaperone GRP78 to low-grade ER stress conditions in young dams. On the contrary, reduces the expression of GRP78 under severe ER stress, which could be beneficial to avoid cells entering into the apoptosis pathway that was evident in aged TUDCA-treated dams.

Further, in aged untreated animals, there were no differences in other ER stress markers aside from GRP78 and we speculate the reason could be due to the fact that we assessed ER stress at late gestation (GD20). It is possible that changes in ER stress markers occurred earlier in pregnancy in aged animals and thus our study missed the gestational window to observe any effects. Assessing earlier gestational ages could be considered for future experiments.

In the female labyrinth zone, an increase in the expression of phospho-eIF2α was observed in aged dams compared to young control, although TUDCA treatment did not significantly reduce the expression of phospho-eIF2α in aged dams, there was a significant interaction (p = 0.023), whereby the aging effect was no longer significant after TUDCA treatment. The higher expression of phospho-eIF2α in female placentas in aged dams suggests that these dams were in the adaptive phase (protective phase) and could have the capacity to restore ER homeostasis via negative feedback dephosphorylation of phospho-eIF2α to terminate the global translation of proteins, and thus consequently, no effect of TUDCA treatment was observed. Novoa *et al*. demonstrated in PERK−/− and GCN2−/− cells that growth arrest and DNA damage protein 34-GADD34 causes dephosphorylation of eIF2α, via negative feedback mechanism and inhibits stress-induced gene expression, thus may promote recovery from translational inhibition of UPR. In addition to ER stress via PERK-mediated eIF2α activation, other conditions such as viral infection (PKR), amino acid starvation (general control non-derepressible-2), and heme deficiency (heme-regulated inhibitor kinase) also phosphorylates eIF2α [65–69], thus, examining all the conditions in the male and female labyrinth and junctional zones were beyond the scope of the current study. We speculate that the reason we did not observe changes in the expression of other ER stress markers (GRP78, ATF-4, CHOP, AFT-6α, and sXBP-1) between young and aged groups was that in the female labyrinth zone there was no activation of the UPR pathway. Previously, we have demonstrated increased oxidative stress in the placenta of female (+57%) and male fetuses (+90%), and increased apoptosis (cleaved caspase 3 via immunohistochemistry) only in the placenta of males from aged dams but not in female placentas [30]. These data also demonstrated that female fetuses of aged dams had beneficial changes in placental transport and endocrine function (up-regulation of insulin-like growth factor 2) compared to young dams, while with male fetuses, there were no beneficial changes in placental transport/hormone expression (reduced insulin-like growth factor 2) in aged dams compared to young dams [30]. Thus, advanced maternal age could alter placental phenotype in a sex-specific manner. As ER and oxidative stress are closely linked with cellular homeostasis and apoptosis, we speculate that it is possible that female placentas are ‘advantageous’ by being less sensitive to ER stress compared to male placentas.

In the male and female junctional zones, there were no changes in the levels of other ER stress proteins (GRP78, phospho-eIF2α, ATF-4, CHOP, AFT-6α, and sXBP-1) observed between the young and aged control groups. Conversely, we observed changes in the labyrinth zone (as discussed in the above paragraph), and we speculate that this may be due to the differences in cell type (labyrinth vs junctional zone). TUDCA treatment reduced the expression of sXBP-1 in both male and female junctional zones compared to aged control, suggesting its effect only on the IRE1α arm of ER stress. Though not in pregnancy, Groenendyk *et al*. in HeartCRT+ (a mouse model of cardiac fibrosis) have demonstrated that TUDCA treatment reduced XBP1 splicing and prevented the activation of IRE1α [70].

Overall, we speculate that the differential expression of ER stress proteins in the labyrinth and junctional zone in placentas from male and female offspring from aged dams may be one of the pathophysiology features that contribute to poor pregnancy outcomes. Although, the exact mechanism by which TUDCA improved fetal body weight in aged dams is not clear, we demonstrated that TUDCA prevented age-induced alterations in placental ER stress markers. Previously we have demonstrated a trend in increased uterine arteries vasodilation response in TUDCA-treated aged dams. Therefore, we speculate that TUDCA also acts independent of the placental effect through improved vascular ER stress in the uterine artery similar to the mesenteric arteries (reduced ER stress proteins: phospho-eIF2α and CHOP) leading to improved maternal adaptations and pregnancy outcomes (i.e., fetal growth) in aged dams.

## Conclusion

Advanced maternal age is associated with an increased risk of fetal growth restriction, preeclampsia, and preterm birth. The current study demonstrated low-grade ER stress with upregulation of GRP78 (in the male labyrinth zone) and phospho-eIF2α (in male and female junctional zones) in aged dams compared to young dams. Further, TUDCA intervention reduced the expression of ER stress markers only in aged dams (GRP78, phospho-eIF2α, ATF-4, and CHOP) in the male labyrinth zone, and sXBP1 protein in both male and female junctional zones. These findings highlight the complexity of cellular stress responses in advanced maternal age, and in particular that ER stress markers may be modulated in aged dams in a sexually dimorphic manner. Furthermore, the results from this study suggest that TUDCA treatment may have a cytoprotective role in the placenta by maintaining ER stress proteins to basal levels.

## Supporting information

S1 Raw images (PDF)
