# Supplementary material for: The effect of tauroursodeoxycholic Acid (TUDCA) treatment on placental endoplasmic reticulum (ER) stress in a rat model of advanced maternal age
Source: PLoS One. 2023 Apr 6;18(4):e0282442. doi: 10.1371/journal.pone.0282442 (PMC10079071; doi:10.1371/journal.pone.0282442)
Supplement: S1 Raw images — (PDF) [file pone.0282442.s001.pdf]

Data Supplement - original Western blots

Fig 2A

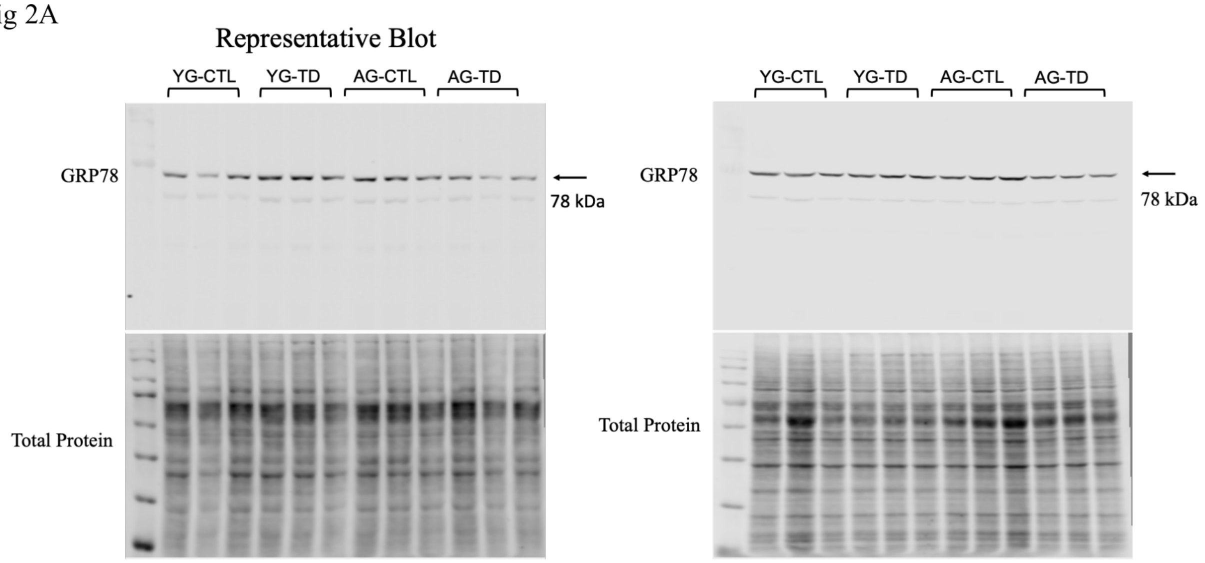

Fig 2B

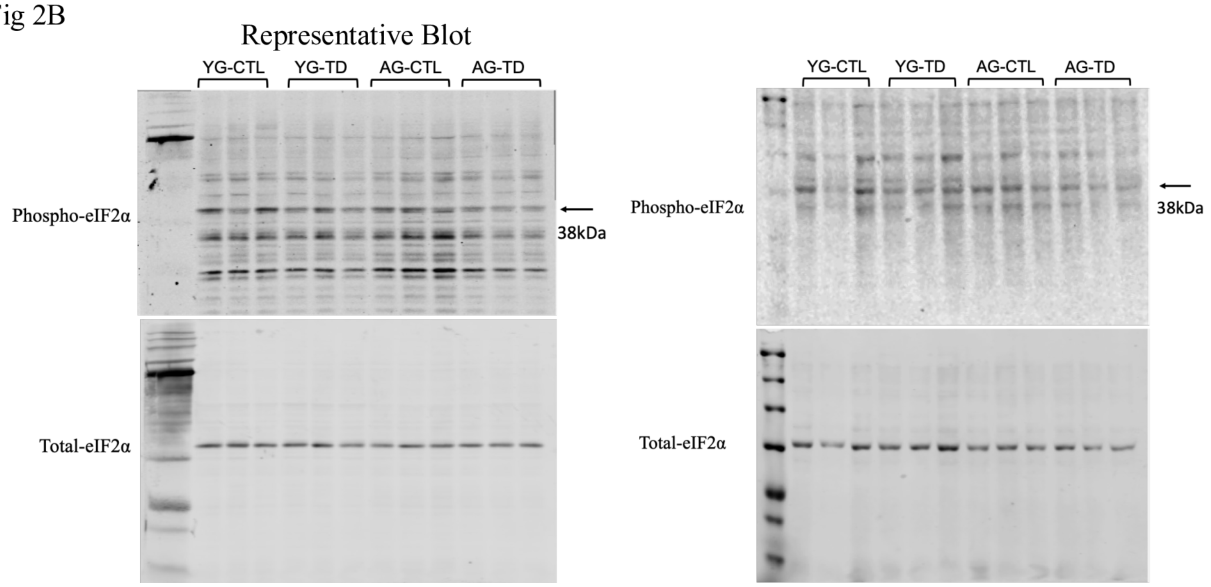

Fig 2C

Representative Blot

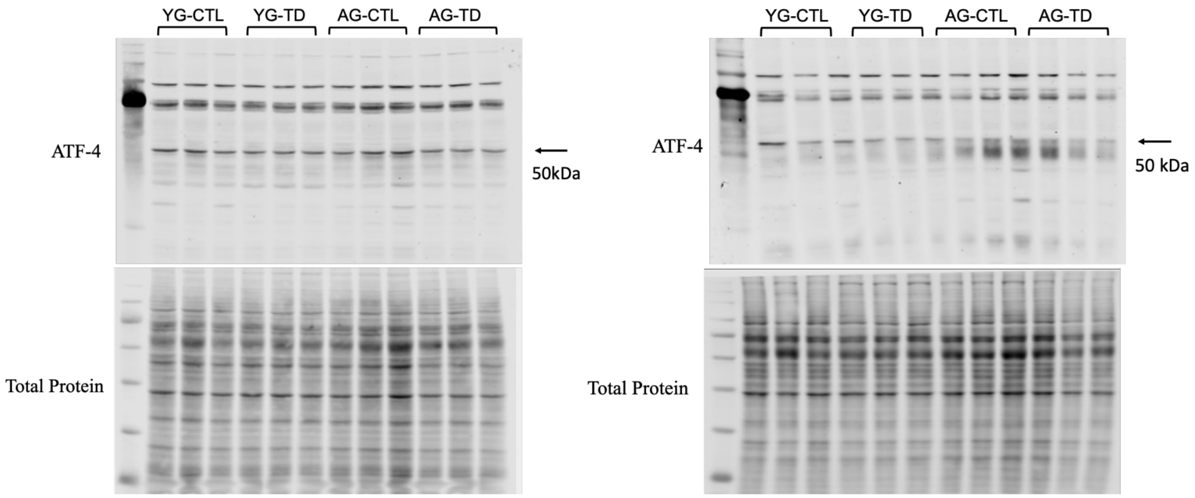

Fig 2D

Representative Blot

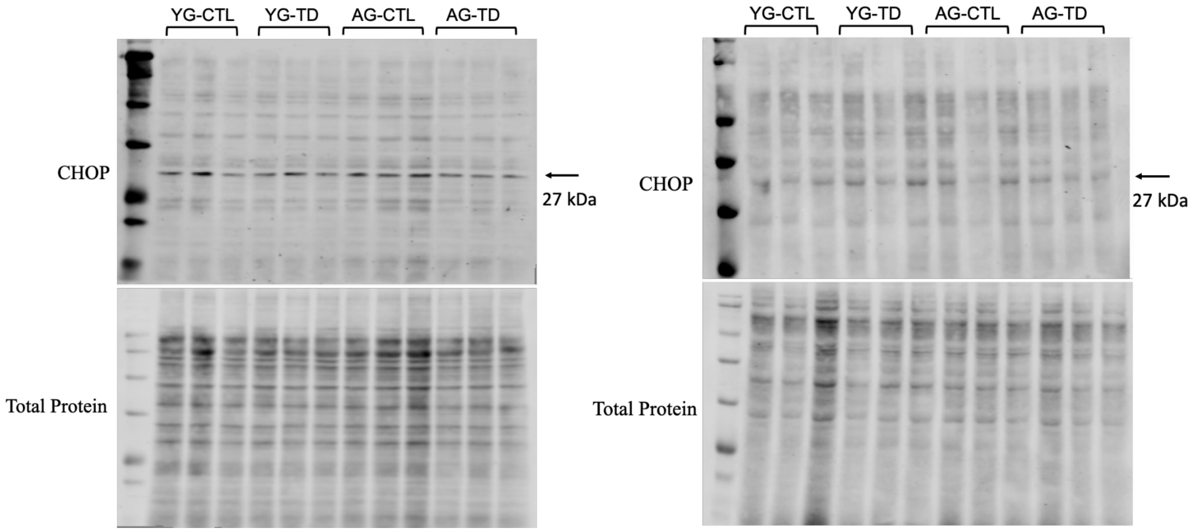

Fig 2E

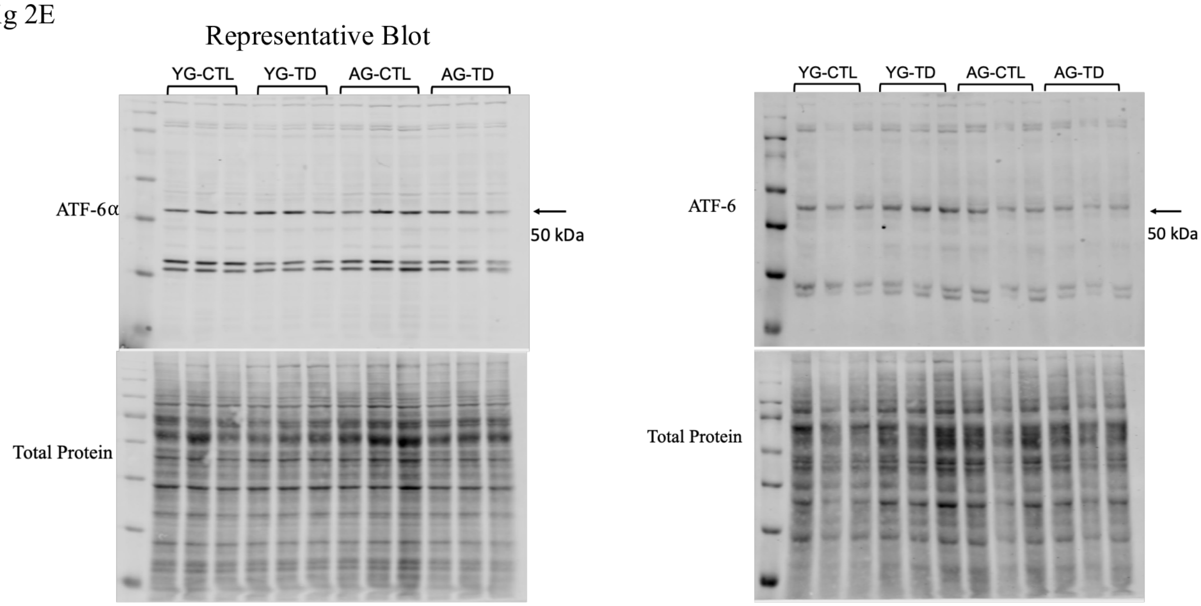

Fig 2F

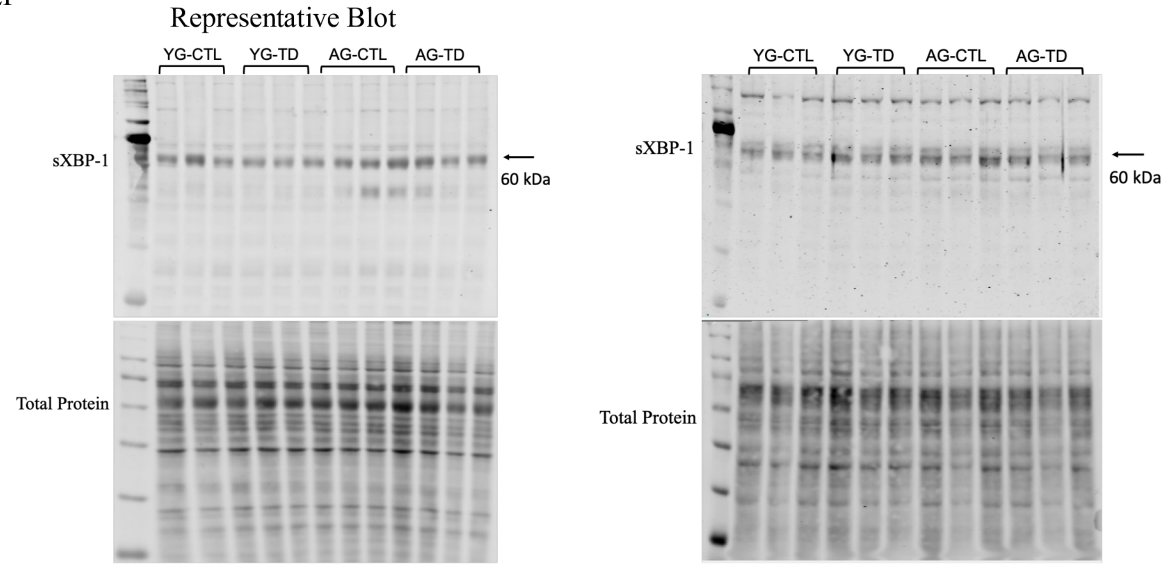

S1 Fig. Expression of ER stress markers in male placental labyrinth zone. Original Western blotting images and images of the total protein staining for GRP78, phospho-eIF2α, ATF-4, CHOP, ATF-6α, and sXBPI protein expression in male placental labyrinth zone offspring from young and aged with or without TUDCA-treatment. Analyzed data is shown in Fig 2. YG- CTL=Young control dams; YG-TD=Young TUDCA-treated dams; AG-CTL=Aged control dams; AG-TD=Aged TUDCA-treated dams.

Fig 3A

Representative Blot

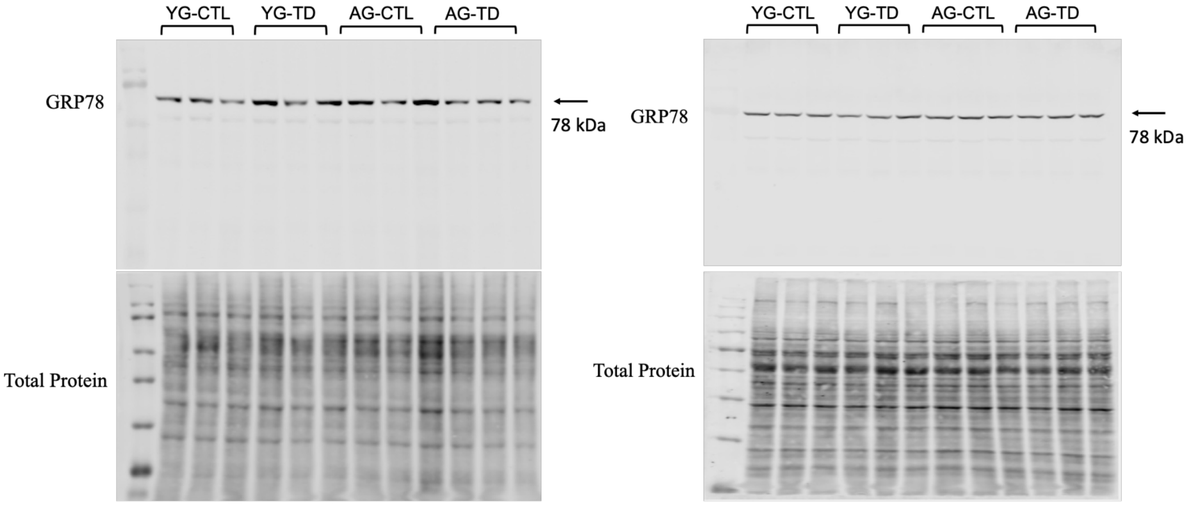

Fig 3B

Representative Blot

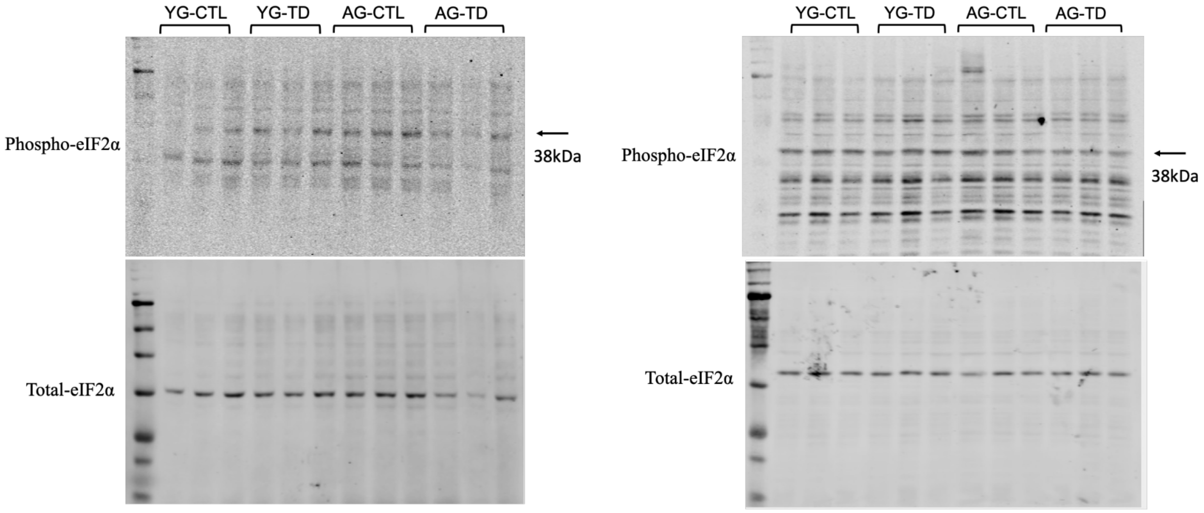

Fig 3C

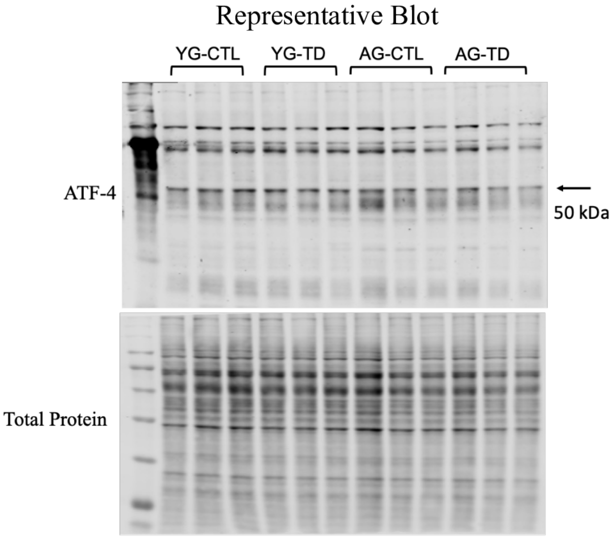
Representative Blot
YG-CTL
YG-TD
AG-CTL
AG-TD
ATF-4
50 kDa
Total Protein

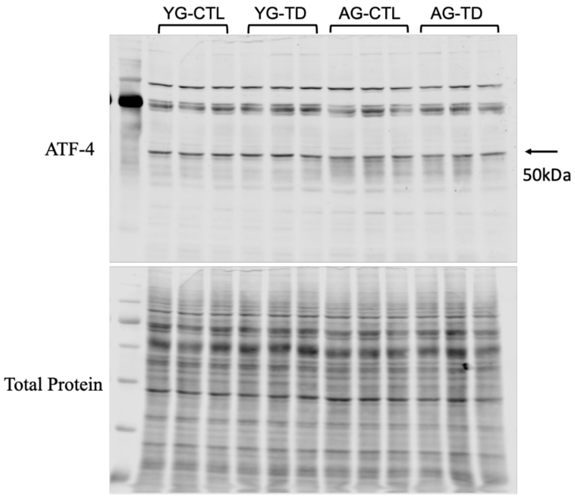
YG-CTL
YG-TD
AG-CTL
AG-TD
ATF-4
50kDa
Total Protein

Fig 3D

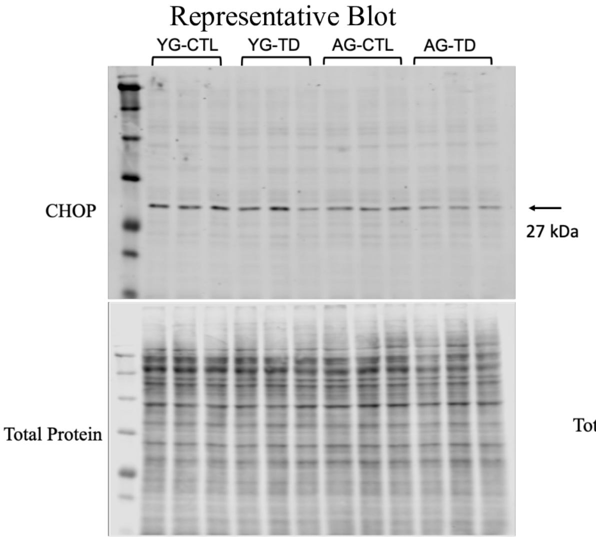
Representative Blot
YG-CTL
YG-TD
AG-CTL
AG-TD
CHOP
27 kDa
Total Protein

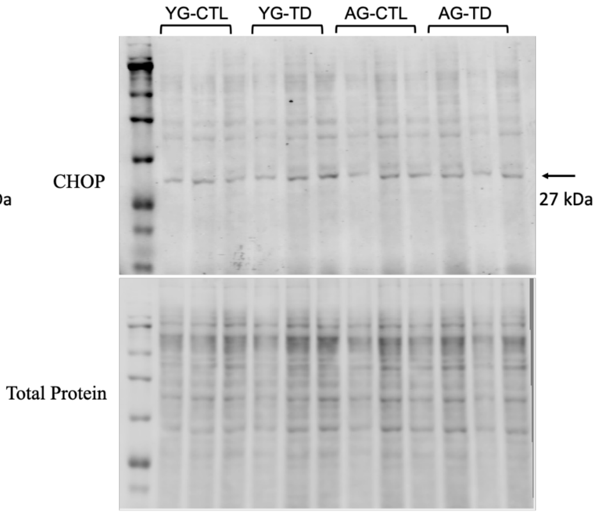
YG-CTL
YG-TD
AG-CTL
AG-TD
CHOP
27 kDa
Total Protein

Fig 3E

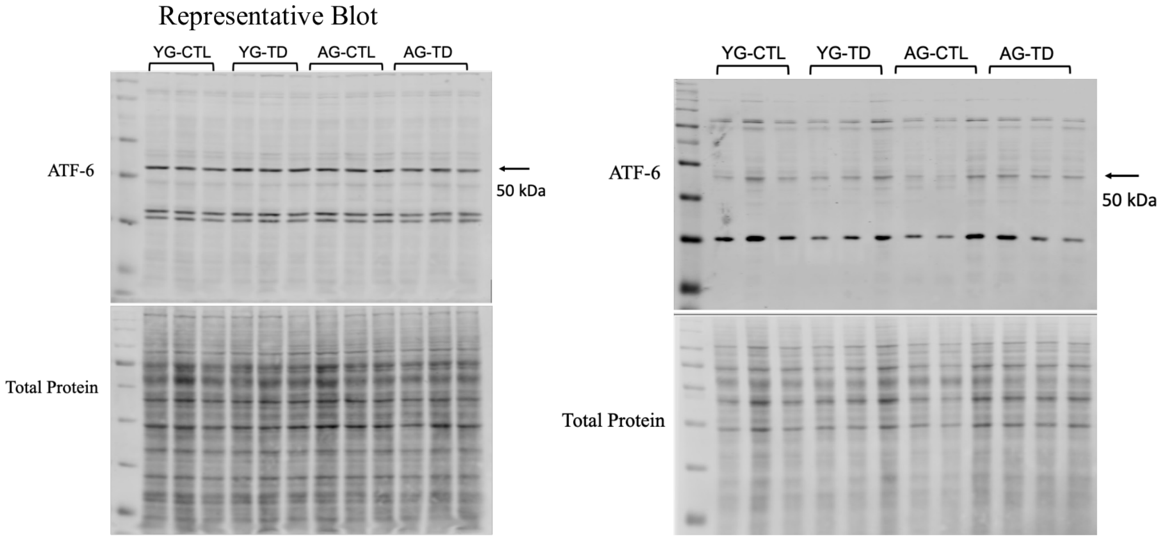

Fig 3F

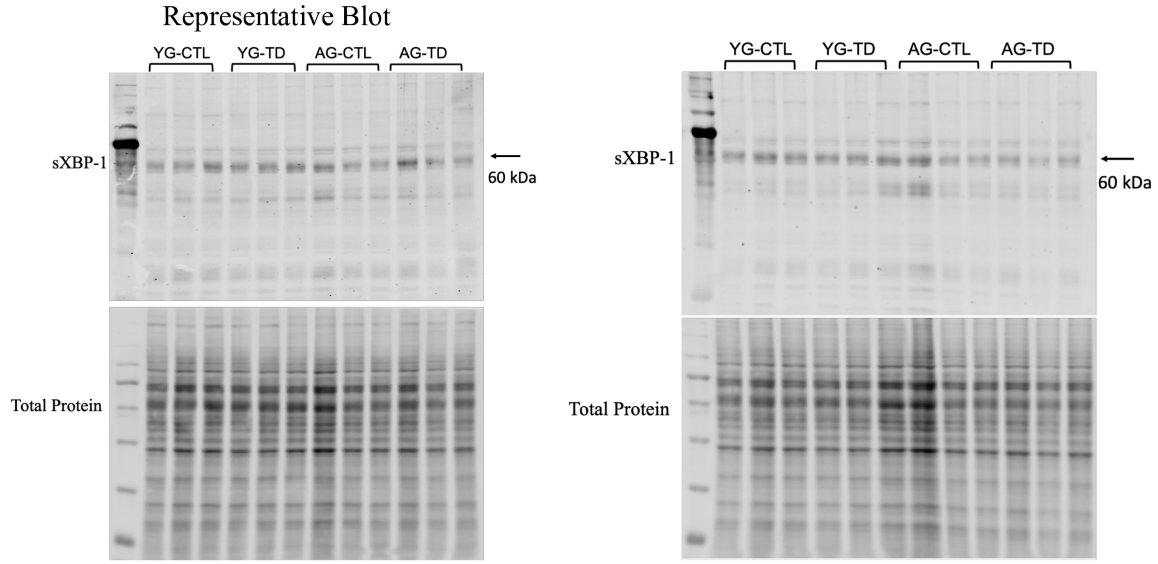

S2 Fig. Expression of ER stress markers in female placental labyrinth zone. Original Western blotting images and images of the total protein staining for GRP78, phospho-eIF2α, ATF-4, CHOP, ATF-6α, and sXBPI protein expression in female placental labyrinth zone offspring from young and aged with or without TUDCA-treatment. Analyzed data is shown in Fig 3. YG- CTL=Young control dams; YG-TD=Young TUDCA-treated dams; AG-CTL=Aged control dams; AG-TD=Aged TUDCA-treated dams.

Fig 4A

Representative Blot

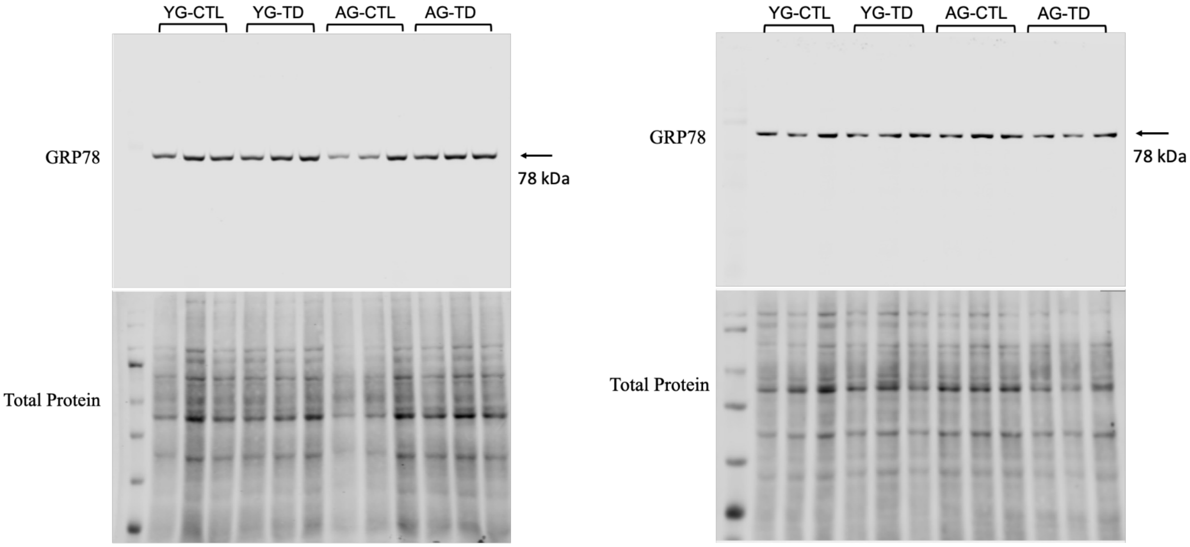

Fig 4B

Representative Blot

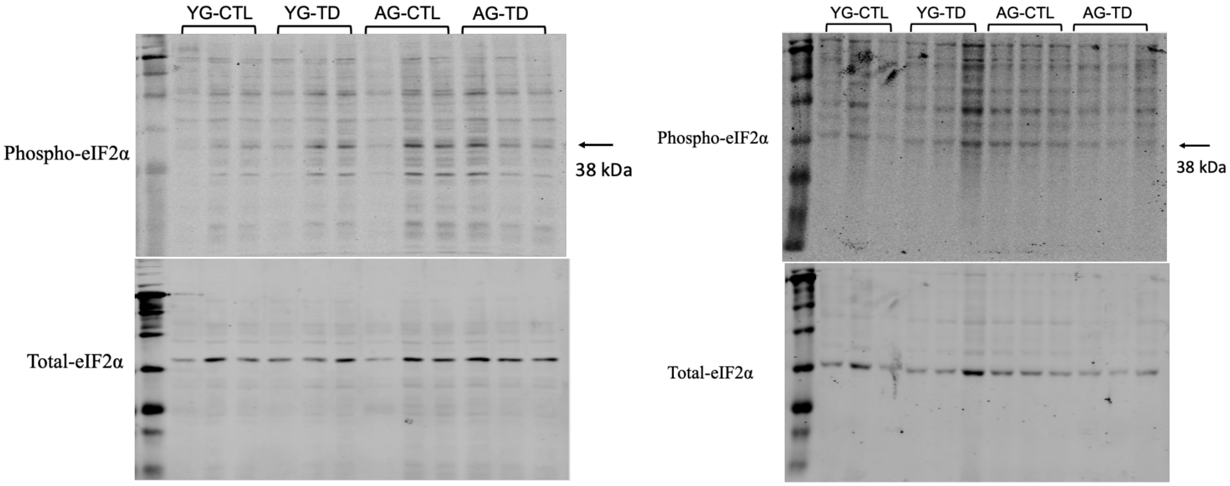

Fig 4C

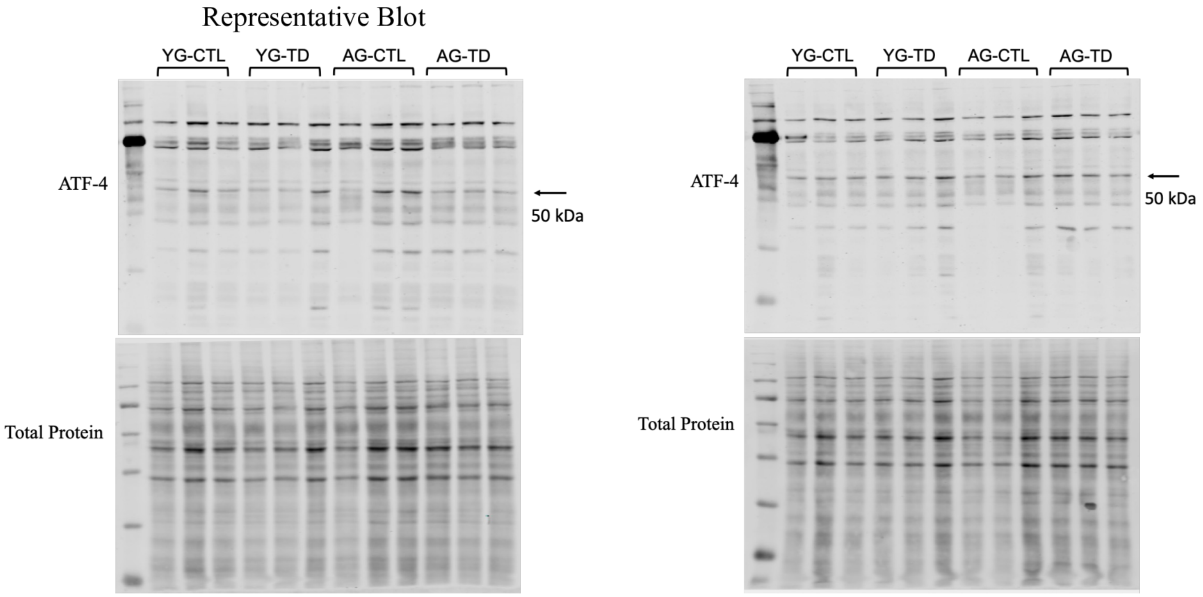

Representative Blot
YG-CTL
YG-TD
AG-CTL
AG-TD
ATF-4
50 kDa
Total Protein
YG-CTL
YG-TD
AG-CTL
AG-TD
ATF-4
50 kDa
Total Protein

Fig 4D

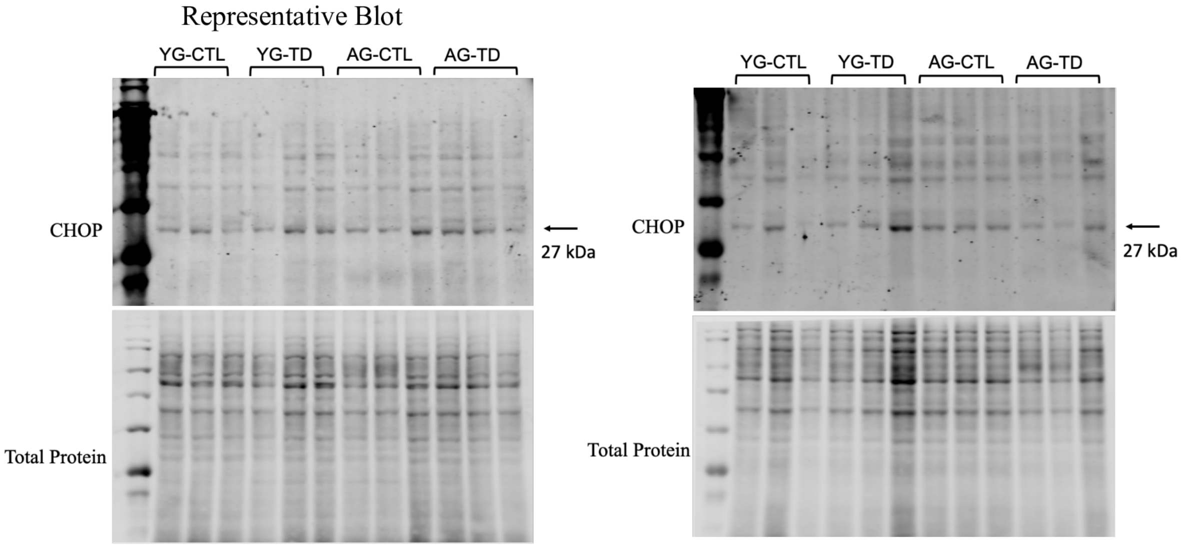

Representative Blot
YG-CTL
YG-TD
AG-CTL
AG-TD
CHOP
27 kDa
Total Protein
YG-CTL
YG-TD
AG-CTL
AG-TD
CHOP
27 kDa
Total Protein

Fig 4E

Representative Blot

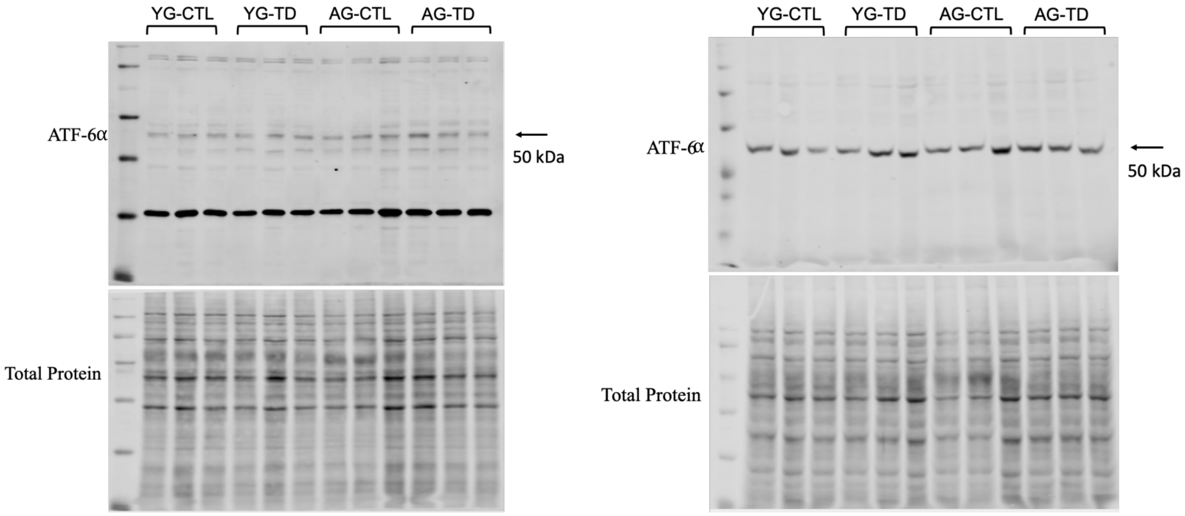

Fig 4F

Representative Blot

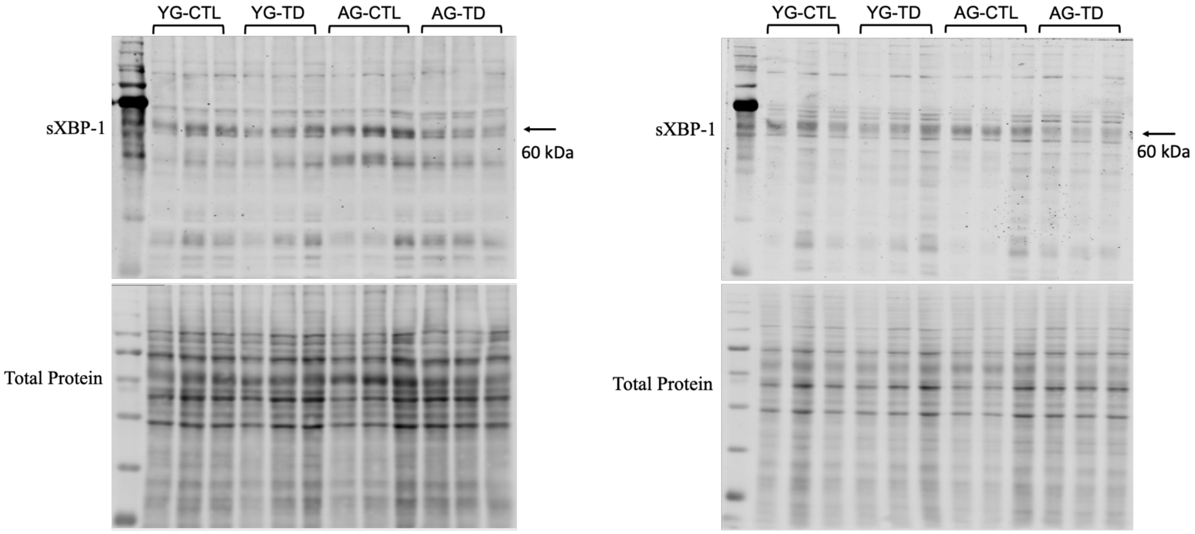

S3 Fig. Expression of ER stress markers in male placental junctional zone. Original Western blotting images and images of the total protein staining for GRP78, phospho-eIF2α, ATF-4, CHOP, ATF-6α, and sXBPI protein expression in male placental junctional zone offspring from young and aged with or without TUDCA-treatment. Analyzed data is shown in Fig 4. YG- CTL=Young control dams; YG-TD=Young TUDCA-treated dams; AG-CTL=Aged control dams; AG-TD=Aged TUDCA-treated dams.

Fig 5A

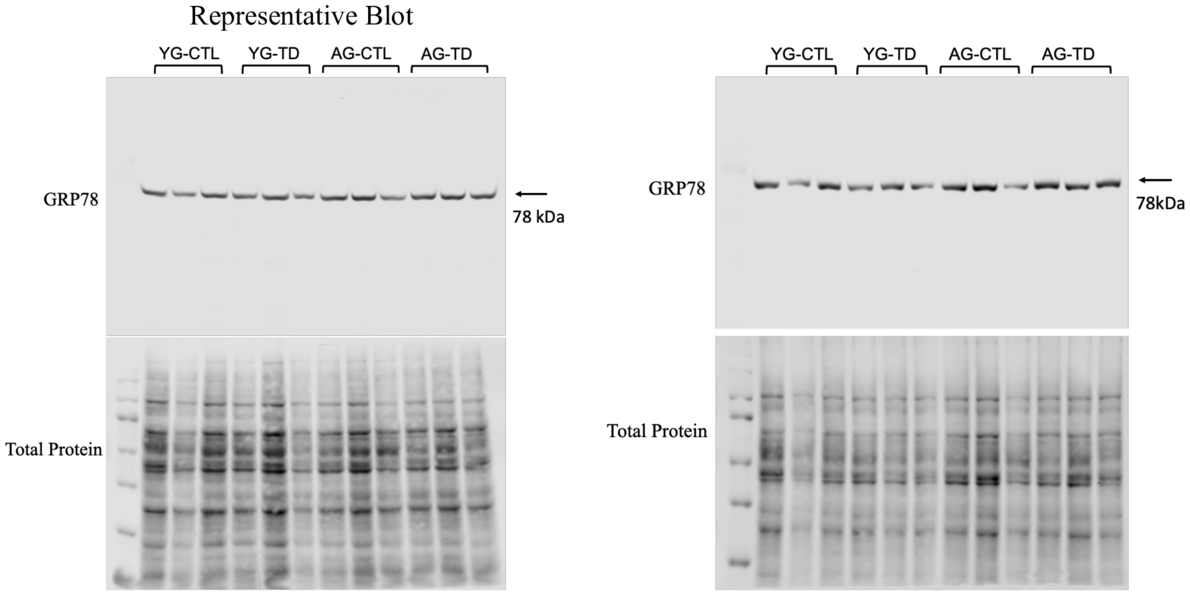

Representative Blot
YG-CTL
YG-TD
AG-CTL
AG-TD
GRP78
78 kDa
Total Protein
YG-CTL
YG-TD
AG-CTL
AG-TD
GRP78
78kDa
Total Protein

Fig 5B

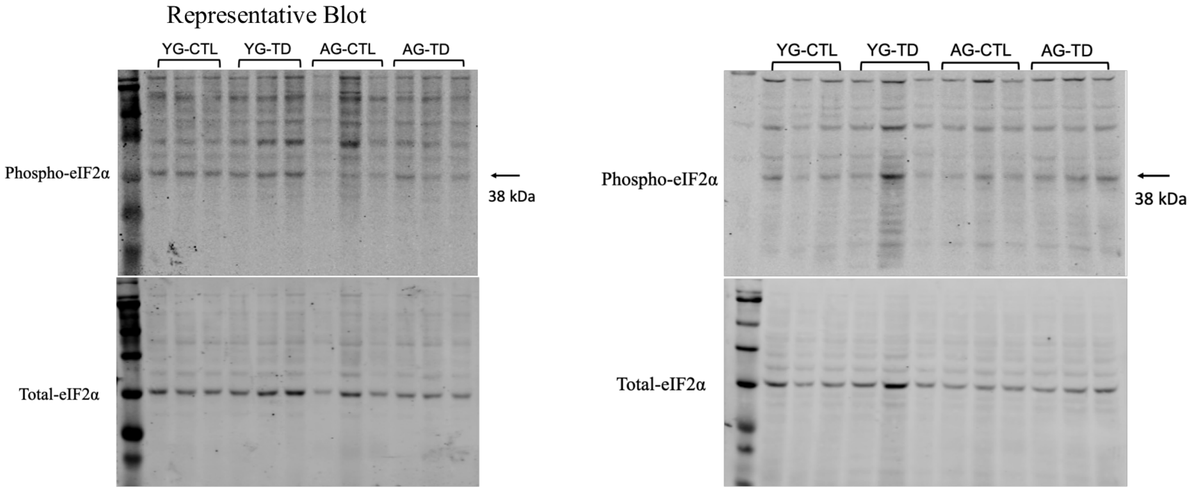

Representative Blot
YG-CTL
YG-TD
AG-CTL
AG-TD
Phospho-eIF2α
38 kDa
Total-eIF2α
YG-CTL
YG-TD
AG-CTL
AG-TD
Phospho-eIF2α
38 kDa
Total-eIF2α

Fig 5C

Representative Blot

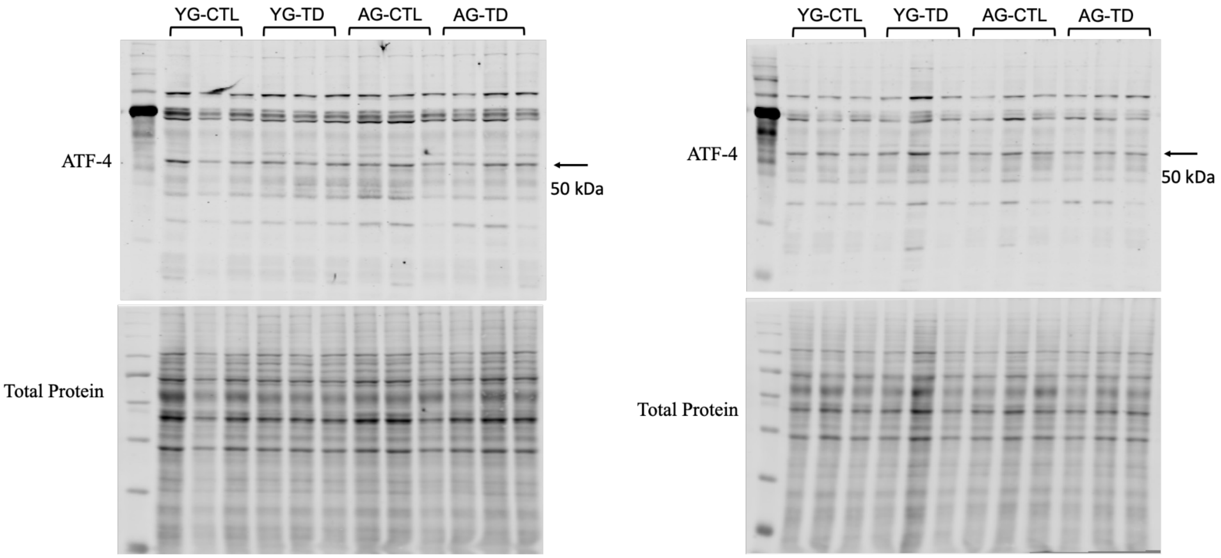

Fig 5D

Representative Blot

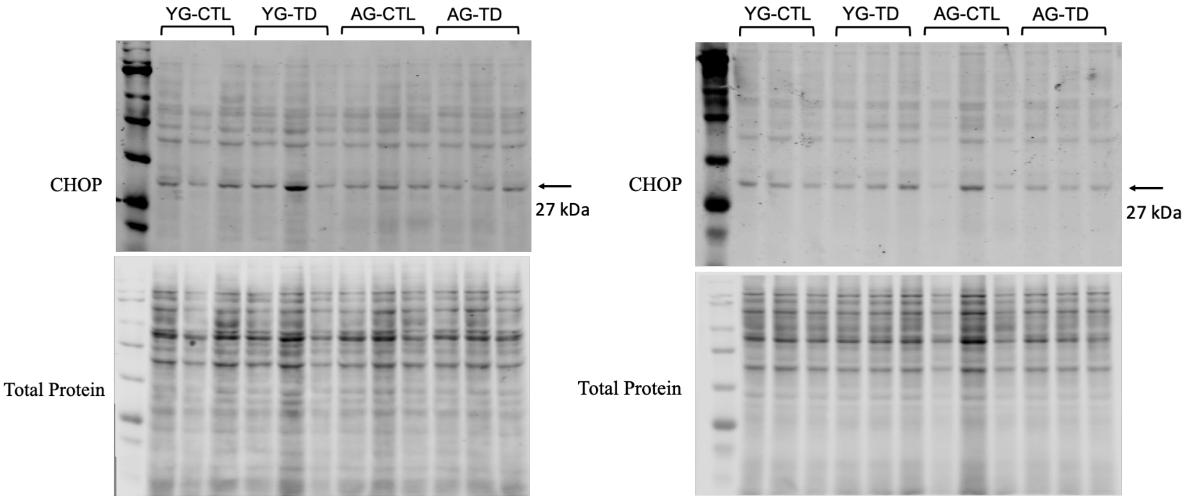

Fig 5E

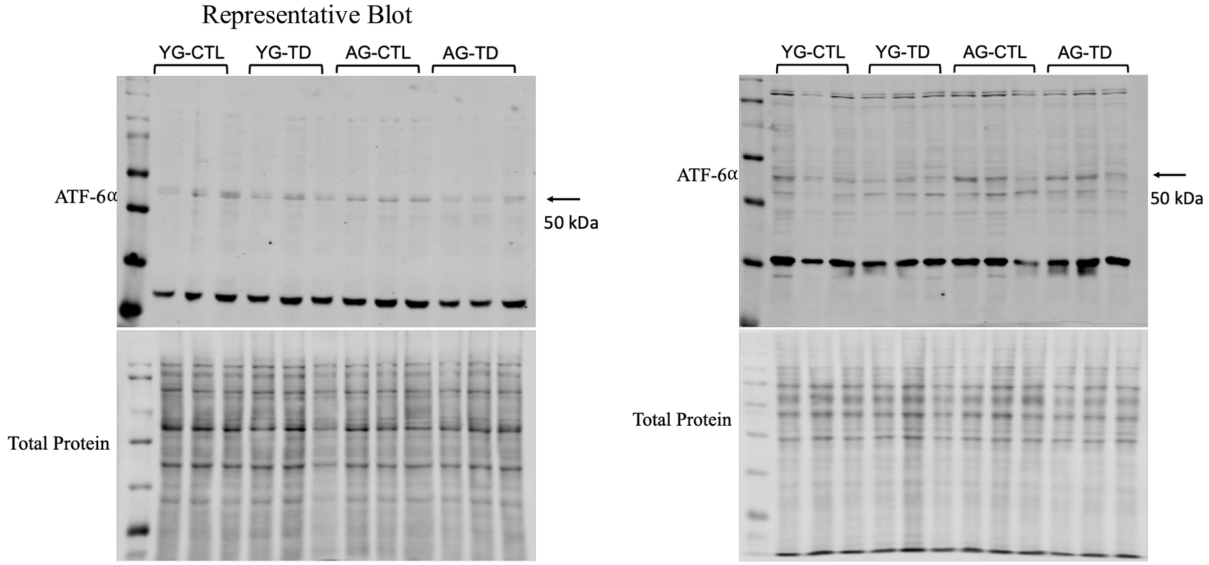

Fig 5F

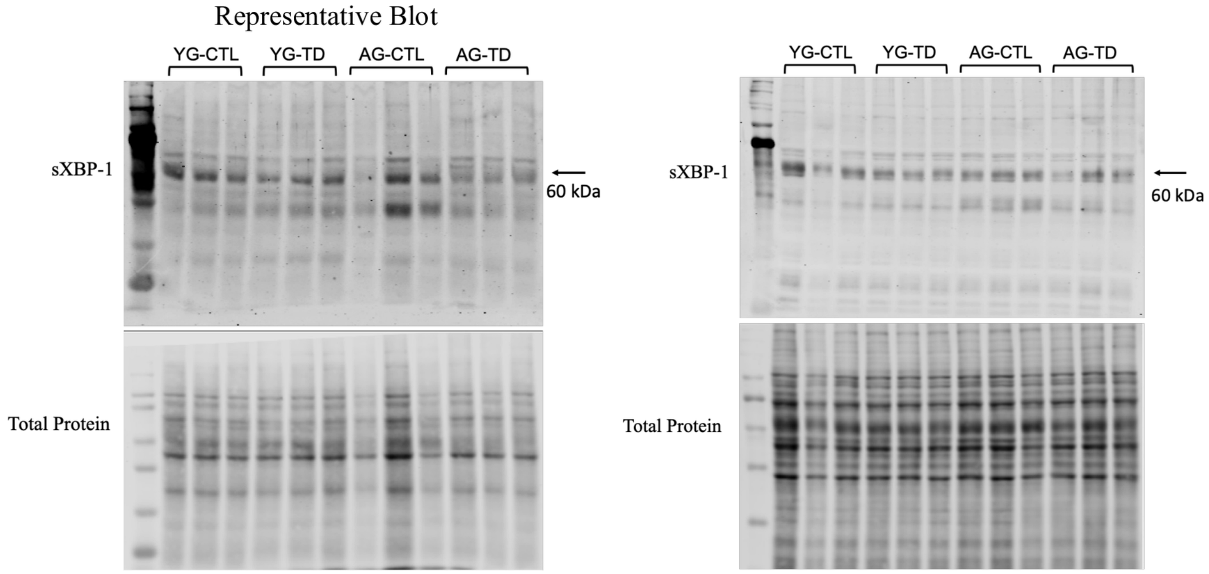

S4 Fig. Expression of ER stress markers in female placental junctional zone. Original Western blotting images and images of the total protein staining for GRP78, phospho-eIF2α, ATF-4, CHOP, ATF-6α, and sXBPI protein expression in female placental junctional zone offspring from young and aged with or without TUDCA-treatment. Analyzed data is shown in Fig 5. YG- CTL=Young control dams; YG-TD=Young TUDCA-treated dams; AG-CTL=Aged control dams; AG-TD=Aged TUDCA-treated dams.
